# Novel Drug Delivery Particles Can Provide Dual Effects on Cancer “Theranostics” in Boron Neutron Capture Therapy

**DOI:** 10.3390/cells14010060

**Published:** 2025-01-06

**Authors:** Abdul Basith Fithroni, Haruki Inoue, Shengli Zhou, Taufik Fatwa Nur Hakim, Takashi Tada, Minoru Suzuki, Yoshinori Sakurai, Manabu Ishimoto, Naoyuki Yamada, Rani Sauriasari, Wolfgang A. G. Sauerwein, Kazunori Watanabe, Takashi Ohtsuki, Eiji Matsuura

**Affiliations:** 1 Graduate School of Interdisciplinary Science and Engineering in Health Systems, Okayama University, Okayama 700-8530, Japan; abdul-bf@s.okayama-u.ac.jp (A.B.F.); kako122232@s.okayama-u.ac.jp (H.I.); p0ux6145@s.okayama-u.ac.jp (S.Z.); pmcx89by@s.okayama-u.ac.jp (T.F.N.H.); pv6s2ev1@s.okayama-u.ac.jp (T.T.); k_watanabe@okayama-u.ac.jp (K.W.); ohtsuk@okayama-u.ac.jp (T.O.); 2 Institute for Integrated Radiation and Nuclear Science, Kyoto University, Osaka 590-0494, Japan; suzuki.minoru.3x@kyoto-u.ac.jp (M.S.); sakurai.yoshinori.8n@kyoto-u.ac.jp (Y.S.); 3 J-BEAM, Inc., Fukushima 979-0513, Japan; manabu.ishimoto@gmail.com; 4 Nihon Fukushi Fuiin Holding, Co., Ltd., Fukushima 979-0513, Japan; naoyuki.yamada@fukushi-chuho.com; 5 Faculty of Pharmacy, Universitas Indonesia, Depok 16424, Indonesia; rani@farmasi.ui.ac.id; 6 Deutsche Gesellschaft für Bor-Neutroneneinfangtherapie DGBNCT e.V., University Hospital Essen, Klinik für Strahlentherapie, 45122 Essen, Germany; wolfgang.sauerwein@dgbnct.de; 7 Collaborative Research Center for OMIC, Graduate School of Medicine, Dentistry, and Pharmaceutical Sciences, Okayama University, Okayama 700-8558, Japan; 8 Neutron Therapy Research Center (NTRC), Okayama University, Okayama 700-8558, Japan

**Keywords:** boron neutron capture therapy (BNCT), dual therapeutic effects, Lactosome^®^, hydrophobic boron compound, neutron irradiation, theranostics

## Abstract

Boron (B) neutron capture therapy (BNCT) is a novel non-invasive targeted cancer therapy based on the nuclear capture reaction ^10^B (n, alpha) ^7^Li that enables the death of cancer cells without damaging neighboring normal cells. However, the development of clinically approved boron drugs remains challenging. We have previously reported on self-forming nanoparticles for drug delivery consisting of a biodegradable polymer, namely, “AB-type” Lactosome^®^ nanoparticles (AB-Lac particles)- highly loaded with hydrophobic B compounds, namely *o*-Carborane (Carb) or 1,2-dihexyl-*o*-Carborane (diC6-Carb), and the latter (diC6-Carb) especially showed the “molecular glue” effect. Here we present in vivo and ex vivo studies with human pancreatic cancer (AsPC-1) cells to find therapeutically optimal formulas and the appropriate treatment conditions for these particles. The biodistribution of the particles was assessed by the tumor/normal tissue ratio (T/N) in terms of tumor/muscle (T/M) and tumor/blood (T/B) ratios using near-infrared fluorescence (NIRF) imaging with indocyanine green (ICG). The in vivo and ex vivo accumulation of B delivered by the injected AB-Lac particles in tumor lesions reached a maximum by 12 h post-injection. Irradiation studies conducted both in vitro and in vivo showed that AB-Lac particles-loaded with either ^10^B-Carb or ^10^B-diC6-Carb significantly inhibited the growth of AsPC-1 cancer cells or strongly inhibited their growth, with the latter method being significantly more effective. Surprisingly, a similar in vitro and in vivo irradiation study showed that ICG-labeled AB-Lac particles alone, i.e., without any ^10^B compounds, also revealed a significant inhibition. Therefore, we expect that our ICG-labeled AB-Lac particles-loaded with ^10^B compound(s) may be a novel and promising candidate for providing not only NIRF imaging for a practical diagnosis but also the dual therapeutic effects of induced cancer cell death, i.e., “theranostics”.

## 1. Introduction

Pancreatic cancer is one of the most aggressive and lethal malignancies, that often presents at an advanced stage when diagnosed and spreads to other parts of the body [1]. The American Cancer Society estimates that approximately 64,050 people (33,130 men and 30,920 women) were diagnosed with pancreatic cancer, and about 50,550 people (26,620 men and 23,930 women) died in the United States [2].

Boron neutron capture therapy (BNCT) is a targeted therapy based on the property of the non-radioactive isotope ^10^B to capture low-energy (<0.5 eV) neutrons with a high effectivity (cross-section: 3835 barns). This nuclear reaction results in an alpha (^4^He) particle and a recoiled lithium nucleus (^7^Li) [3,4]. Given their short path lengths in tissues (5–9 μm), BNCT theoretically allows selectively killing malignant boron-containing cells while sparing adjacent normal cells without boron uptake [5].

For successful BNCT treatment, the following criteria are required: (1) selective uptake of ^10^B-containing agents by tumor cells at concentrations sufficiently high to deliver a therapeutic dose of ^10^B atoms (≥20 μg ^10^B/g of tumor tissue (lesion) or 10^9^ atoms of ^10^B/cancer cell) as mentioned by Kueffer, et al.; (2) high retention of ^10^B in tumor tissues together with high clearance from blood and normal tissues; and (3) low systemic toxicity [6]. ^10^B-boronophenylalanine (^10^B-BPA) and ^10^B-sodium borocaptate (^10^B-BSH) are the first generation of B compounds clinically applied. However, neither ^10^B-BPA nor ^10^B-BSH fully meets these criteria in an optimal way. Nevertheless, these compounds were selected because they were the best available options and could provide us with the benchmark to evaluate the novel boron drugs for BNCT [7]. Due to these limitations, significant efforts have been expended to develop novel boron delivery agents with more favorable biodistribution and uptake, including boron-containing porphyrins, amino acids, polyamines, nucleosides, peptides, monoclonal antibodies, liposomes, various types of nanoparticles, B compounds, and copolymers [8].

For B agents used in conjunction with drug delivery systems (DDSs) such as nanoparticles, polyhedral B compounds are more attractive for development compared with those with a single B atom per molecule. Among the newly discovered compounds, the highly hydrophobic “carborane (Carb)”, which contains two carbon atoms and ten B atoms, has been explored. It exists in individual three isoforms characterized by substituents of *ortho (o) (1, 2)*, *meta (m) (1, 7)*, or *para (p) (1, 12)* positions in their structure [9].

One of the promising self-forming nanoparticles, which is made of a biodegradable polymer whose size can be controlled and that can be easily modified for targeting and imaging, is the “AB-type Lactosome^®^” nanoparticle (AB-Lac particle) [10]. These biodegradable nanoparticles consist of an amphipathic polydepsipeptide, which is a hydrophilic polysarcosine (PSar) chain, “A”, linked to another hydrophobic poly-L-lactic acid (PLLA) chain, “B”. This polymer assembly forms micelle-like particles by itself in an aqueous phase and can be easily degraded by hydrolysis under physiological conditions. The physical properties of the AB-Lac polymer PSar_64_-PLLA_30_, was reported to be hydrodynamic diameter: ca. 36 nm and blood half-life: 17.2 h [11]. In the present study, we used a slightly longer AB-Lac-Polymer, PSar_106_-PLLA_32_ (67 nm diameter), to achieve a much more efficient “enhanced permeability and retention (EPR)” effect, but the blood half-life should be similar.

Recently, the utilization of the EPR effect as a tumor-targeting delivery system has been widely used in the therapeutic modalities, diagnostic imaging, and “theranostics”, combining therapy and diagnosis [12,13]. Recent BNCT research has explored theranostic approaches using modalities such as PLGA nanoparticles [14], PLGA-*block*-PEG nanoparticles [15], and *aza*-BODIPY [16].

We developed candidates for novel medicines for BNCT by loading AB-Lac particles with a hydrophobic ^10^B compound. The AB-Lac particles were loaded with three carborane isomers, i.e., *o*-Carb, *m*-Carb, and *p*-Carb, and with three *o*-Carb acyl-derivatives: (1,2-dimethyl-*o*-Carb (diC1-Carb); 1,2-dihexyl-*o*-Carb (diC6-Carb); and 1,2-didodecyl-*o*-Carb (diC12-Carb). In a previous study, we discovered serendipitously the distinctive interaction between AB-Lac particles and one of the *o*-Carb acyl-derivatives, diC6-Carb, namely, the “molecular glue” effect [17].

**Figure 1:**
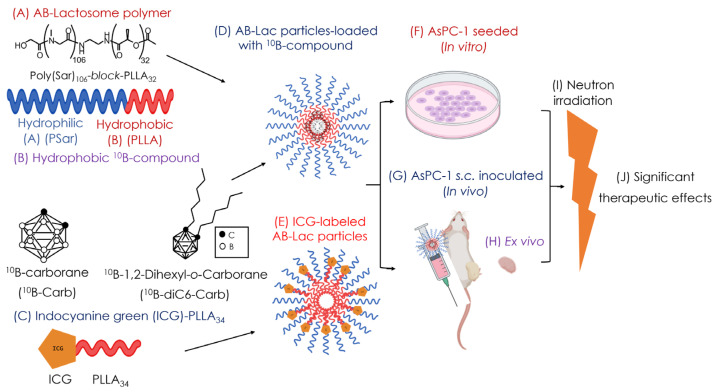
The schematic concepts of the present study. The structure of the AB-Lactosome^®^ polymer (**A**); hydrophobic B compound (^10^B-Carb) and *o*-Carb acyl-derivative (^10^B-diC6-Carb) (**B**); ICG-PLLA (**C**). Schematic illustration of AB-Lac particles-loaded with a hydrophobic ^10^B compound (**D**) and ICG-labeled AB-Lac particles (**E**). Illustration of in vitro (**F**), in vivo (**G**), and ex vivo experiments (**H**) and neutron irradiation (**I**), and therapeutic effects by irradiation were analyzed (**J**).

In the present study (as shown in Figure 1), we evaluated the therapeutic effect of our newly developed ^10^B compounds, AB-Lac particles-loaded with ^10^B-Carb or with ^10^B-diC6-Carb, by performing an in vitro and in vivo neutron irradiation study. In addition, dual effects were fortuitously observed on AB-Lac particles that were labeled with indocyanine green (ICG) (but without any ^10^B compounds loaded).

## 2. Materials and Methods

### 2.1. Reagents

All reagents were purchased commercially and were of reagent grade. The AB-Lac polymer, polysarcosine (PSar)_106_-*block*-poly-L-lactic acid (PLLA)_32_ (molecular weight: 10,001 Da) (Figure 1A), was synthesized by KNC Laboratories, Co., Ltd. (Kobe, Japan). ^10^B-Carb and ^10^B-diC6-Carb (Figure 1B) were purchased from Katchem, Ltd. (Prague, Czech Republic). ^10^B-BPA was purchased from Interpharma Praha, a.s. (IPP) (Prague, Czech Republic). ICG NHS ester ® was purchased from BioActs (Incheon, Korea), NH2 PLLA was purchased from (Xi’an Ruixi Biological Technology, Co., Ltd., Shaanxi, China), RPMI-1640 medium, Dulbecco’s modified phosphate-buffered saline (DPBS), crystal violet (CV), chloroform, hydrogen peroxide (H_2_O_2_), and perchloric acid (HClO_4_) were purchased from Fujifilm-Wako Pure Chemical Corp. (Osaka, Japan). Fetal bovine serum (FBS) was purchased from Sigma-Aldrich, Co., Ltd. (St. Louis, MO, USA). Corning Matrigel^®^ was purchased from Corning Life Sciences (Bedford, MA, USA). B standard solution, penicillin–streptomycin mixed solution, and the trypsin (2.5 g/L)-EDTA (1 mmol/L) solution were purchased from Nacalai Tesque, Inc. (Kyoto, Japan). The anesthetizers medetomidine hydrochloride (Dorbene Vet^®^) and atipamezole (Atipame^®^) were purchased from Kyoritsu Seiyaku Corp. (Tokyo, Japan), and butorphanol tartrate (Vetorphale^®^) was purchased from Meiji Seika Pharma Co., Ltd. (Tokyo, Japan). Isoflurane was purchased from Viatris Pharmaceutical, Co., Ltd. (Tokyo, Japan). Saline was purchased from Otsuka Pharmaceutical, Co., Ltd. (Tokushima, Japan). CCK-8 kit was purchased from Dojindo Molecular Technologies, Inc. (Kumamoto, Japan).

### 2.2. Cell Culture

Human pancreatic cancer (AsPC-1) cells were purchased from the American Type Culture Collection (ATCC) (Rockville, MD, USA), and they were maintained in RPMI-1640 medium supplemented with 10% (*v*/*v*) of FBS and 1% (*v*/*v*) of the penicillin–streptomycin solution under 5% CO_2_ atmosphere at 37 °C. These cells were detached by trypsin-EDTA solution. This method was previously described [17].

### 2.3. Preparation of the B (^10^B)-Compound-Loaded AB-Lac Particles

The AB-Lac particles, PSar_106_-*block*-PLLA_32_ (1 µmol), and the ^10^B compound (10 µmol) were mixed in chloroform (1.1 mL) to form a polymer–^10^B compound film by evaporation. Then, DPBS (2.0 mL) was added and firmly sonicated for 20 min. The nanoparticle-enriched eluate was collected after passing through a PD-10 (GE-Healthcare, Buckinghamshire, UK). The eluate was filtered by a 0.22 µm syringe filter (Merck Millipore, Dublin, Ireland) and a 0.1 µm syringe filter (Pall Corporation, Port Washington, NY, USA) to exclude larger-sized and aggregated particles (Figure 1B,D). The preparation methods (referred to in Methods) were previously reported by Makino, et al. [18]. ^10^B-Carb or ^10^B-diC6-Carb was loaded and composed into AB-Lac particles, and the B amount in the particles was measured with inductively coupled plasma atomic emission spectrophotometry (ICP-AES) (Shimadzu, Kyoto, Japan). The particle size distribution (PSD) and polydispersity index (PDI) of AB-Lac particles were determined by Zetasizer^®^ (Nano ZSP; Malvern, Instruments, Malvern, UK). The PSD and PDI data are represented as mean ± S.D. Surface properties of AB-Lac particles were observed using a transmission electron microscope (TEM) (H-7650, Hitachi High-Tech Solutions, Co. Ltd., Tokyo, Japan) at accelerated voltage at 80 kV and 10,000× magnification. This method was previously described [17].

### 2.4. Preparation of the ICG-Labeled AB-Lac Particles

PSar_106_-*block*-PLLA_32_ (1 µmol) and 1 mol.% of ICG-PLLA_34_ in chloroform (1 mL) were mixed together and evaporated to form a film followed by resuspending in DPBS (2.0 mL) and sonicating for 20 min. The suspension was passed through a PD-10 column to obtain nanoparticle-enriched eluate. The eluate was filtered through the 0.22 and 0.1 µm syringe filters to exclude larger-sized and aggregated particles (Figure 1C,E). Optical density (OD) at 794 nm of suspensions was measured by a BioSpec^®^ spectrometer (Shimadzu, Kyoto, Japan). For labeling with a hydrophilic chemical, e.g., ICG, we covalently esterified NH2-PLLA to a carbonylated chemical. The conjugate has an amphipathic property, and it is easy to incorporate into the particles without any changes in their properties. This method was previously described [17].

### 2.5. Xenograft

Six-week-old female nude mice (BALB/c nu/nu) were purchased from Charles River (Yokohama, Japan). Two weeks prior to the intravenous (i.v.) treatment and/or irradiation, AsPC-1 cells (2 × 10^6^ cells) suspended in DPBS (100 µL) with 40% Corning Matrigel^®^ were subcutaneously (s.c.) inoculated into the right thigh. The body weight and tumor size were monitored every 2–3 days. All animal experiments were approved by the Animal Care and Use Committee of Okayama University (OKU-2022691). Under the ethical regulation, we usually try to shorten the period of painful in vivo study as much as we can, because we have to respect the “Animal Welfare” perspective.

### 2.6. Ex Vivo Biodistribution of ^10^B Compound-Loaded AB-Lac Particles

The AB-Lac particles-loaded with ^10^B-Carb or with ^10^B-diC6-Carb (at a respective ^10^B-dose of 5 mg ^10^B/kg) were intravenously (i.v.) injected via the tails of the respective AsPC-1 cells-bearing mice (xenografts; 4 mice in each group). After that, the mice organs were excised, weighed, digested, and filtered, followed by measurement of ^10^B amount by ICP-AES analysis. This method was previously described [17].

### 2.7. In Vivo and Ex Vivo NIRF Imaging

The ICG-labeled AB-Lac particles (100 µL) or those loaded with ^10^B compounds were i.v. injected via the tail veins of the respective xenografts. The in vivo NIRF imaging was captured at 0, 1, 3, 6, 12, 24, 48, or 72 h after the injection. At 72 h after the imaging, organs were excised, weighed, and imaged. This method was previously described [17].

### 2.8. In Vitro Cytotoxicity

AsPC-1 cells (25 × 10^3^ cells/well) were seeded in a 96-well microplate and cultured under 5% CO_2_ at 37 °C for 24 h prior to the treatment. Subsequently, the cells were treated with AB-Lac particles-loaded with ^10^B-Carb or ^10^B-diC6-Carb at concentrations of 0.050, 0.25, 0.50, 1.0, 2.0, and 5.0 mM (as ^10^B amount equivalent) for 24 h. The cell cytotoxicity test was assessed using the CCK-8 kit, according to the kit’s instructions. The OD at 450 nm was measured in quadruplicate (*n* = 4). This method was previously described [17].

### 2.9. In Vitro Irradiation (For BNCT)

AsPC-1 cells (1.0 × 10^6^ cells/well) were seeded in a 6-well plate and cultured under 5% CO_2_ at 37 °C for 24 h prior to the treatment. The cells were then treated with AB-Lac particles and AB-Lac particles-loaded with ^10^ B-Carb and ^10^B-diC6-Carb (at 0.5 mM and 2.0 mM of B-equivalent, respectively) for 2 h (Figure 1F). Control group was treated with medium and DPBS only. The cells were irradiated by a nuclear reactor (at the Kyoto University Reactor (KUR)) at 1 MW for 10 min (thermal neutron fluence: 1.3 × 10^12^ neutrons/cm^2^, γ-ray dose 0.17 Gy) and 40 min (thermal neutron fluence: 5.6 × 10^12^ neutrons/cm^2^, γ-ray dose: 0.58 Gy), respectively. After the irradiation, the cells were seeded in a 12-well plate (*n* = 4) and cultured under 5% CO_2_ at 37 °C for 14 days. Then, all cultured cells were stained with 0.5% CV in 20% methanol. The colony formation was quantified by ColonyArea^®^ plug-in provided by Image J National Institutes of Health, Bethesda, MD, USA). The colony percentage was calculated on the threshold and area at the selected regions. The quantification was performed with the method previously reported by Ueda, et al. and Guzman, et al. [19,20]. Data were indicated as mean ± S.E.M. Significant differences were represented by ** *p* < 0.01 and * *p* < 0.05.

### 2.10. In Vivo Irradiation (For BNCT)

Fourteen days after the inoculation, the xenografts (4 tumor-bearing mice in each group) were i.v. injected with AB-Lac particles-loaded with ^10^B-Carb or loaded with ^10^B-diC6-Carb (5 mg of ^10^B/kg), respectively (Figure 1G,H). “Cold” control group consisted of the mice without any treatment after cancer cell inoculation, while “hot“ control group consisted of neutron-irradiated mice without injection of any AB-Lac particles. The therapeutic effect of AB-Lac particles-loaded with ^10^B-Carb or with ^10^B-diC6-Carb for BNCT was assessed. Neutron irradiation was performed 24 h after the injection at KUR reactor with 5 MW for 10 min (thermal neutron fluence: 2.5 × 10^12^ neutrons/cm^2^, γ-ray dose: 0.33 Gy) or 40 min (thermal neutron fluence: 9.4 × 10^12^ neutrons/cm^2^, γ-ray dose: 1.2 Gy), respectively. Body weight and tumor size were monitored until 24 days after the irradiation. Data were indicated as mean ± S.E.M. Significant differences were represented by ** *p* < 0.01 and * *p* < 0.05.

### 2.11. In Vitro Irradiation for Inducing Cell Death (Via ICG)

AsPC-1 cells (1.0 × 10^6^ cells/well) were seeded in a 6-well plate and incubated under 5% CO_2_ at 37 °C for 24 h prior to the treatment. Subsequently, the cells were cultured with ICG-labeled AB-Lac particles or AB-Lac particles for 2 h (Figure 1F). The cells were irradiated by a KUR reactor at 1 MW for 10 min (thermal neutron fluence: 1.1 × 10^12^ neutrons/cm^2^, γ-ray dose: 0.14 Gy) and 40 min (thermal neutron fluence: 3.9 × 10^12^ neutrons/cm^2^, γ-ray dose: 0.56 Gy), respectively. After the irradiation, the cells were seeded into a 12-well plate (*n* = 4) and cultured under 5% CO_2_ at 37 °C for 14 days. Then, cells were stained with 0.5% CV in 20% methanol. Colonies were counted using an aCOLyte^®^ 3 automatic colony counter (Synbiosis, A Division of Synoptics Ltd. Cambeidge, UK). Data were indicated as mean ± S.E.M. Significant differences were represented by ** *p* < 0.01 and * *p* < 0.05.

### 2.12. In Vivo Irradiation for Inducing Cell Death (Via ICG)

AsPC-1 tumor-bearing mice (xenografts; 6 mice in each group) were i.v. injected with 100 μL of ICG-labeled AB-Lac particles (10 μM of ICG) after 18 days inoculation as irradiated and non-irradiated groups (Figure 1G,H). The “cold” control was a group without any treatment after tumor inoculation, while ”hot” control was a neutron-irradiated group without injection of any AB-Lac particles. The drugs were i.v. injected into the xenografts at 24 h before irradiation. The irradiation was performed by a KUR reactor at 5 MW for 40 min (thermal neutron fluence: 9.7 × 10^12^ neutrons/cm^2^, γ-ray dose: 0.98 Gy) at the KUR. Tumor size and body weight were monitored until 18 days after irradiation. Data were indicated as mean ± S.E.M. Significant differences were represented by ** *p* < 0.01 and * *p* < 0.05.

## 3. Results

### 3.1. Characterization of AB-Lac Particles-Loaded with ^10^B Compounds

**Figure 2:**
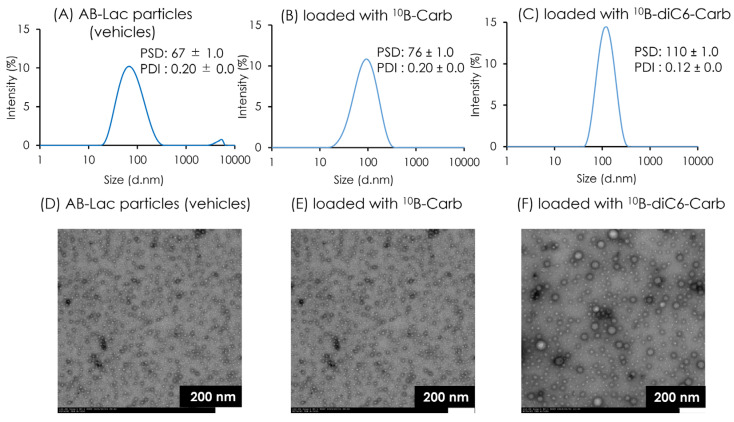
The characterization of AB-Lac particles-loaded with ^10^B compounds. The PSD and PDI of AB-Lac particles (**A**), those loaded with ^10^B-Carb (**B**), and those loaded with ^10^B-diC6-Carb (**C**). TEM images of AB-Lac particles (**D**), those loaded with ^10^B-Carb (**E**), and those loaded with ^10^B-diC6-Carb (**F**) at accelerated voltage at 80 kV and 10,000× magnification. The PSD and PDI are indicated as mean ± S.D. (*n*=3). This result confirmed the results of the previous experiment [17].

The physical properties and morphologies of the AB-Lac particles were analyzed using Zetasizer^®^ and TEM. The PSD of the AB-Lac particles (vehicles) was 67 ± 1.0, while a similar PSD was observed in those loaded with ^10^B-Carb to 76 ± 1.0. However, those loaded with ^10^B-diC6-Carb had the larger PSD, 110 ± 1.0 (Figure 2A–C). Furthermore, all AB-Lac particles showed a similar monodispersity with PDI: 0.1–0.2. The observation of AB-Lac particles by TEM images indicated spherical shapes (Figure 2D–F), which was confirmed by the measurements of PSD by Zetasizer^®^.

### 3.2. Ex Vivo B Biodistribution of ^10^B Compound-Loaded AB-Lac Particles in AsPC-1 Tumor-Bearing Mice for 24 h Post-Injection

The B biodistribution in each organ was analyzed. The AB-Lac particles loaded with ^10^B-Carb or ^10^B-diC6-Carb (5 mg of B-equivalent/kg) were i.v. injected via the tail vein. The mice were euthanized and sacrificed at 24 h after injection. The amount of B in each organ was measured by ICP-AES after digestion with HClO_4_:H_2_O_2_ (1:1).

**Figure 3:**
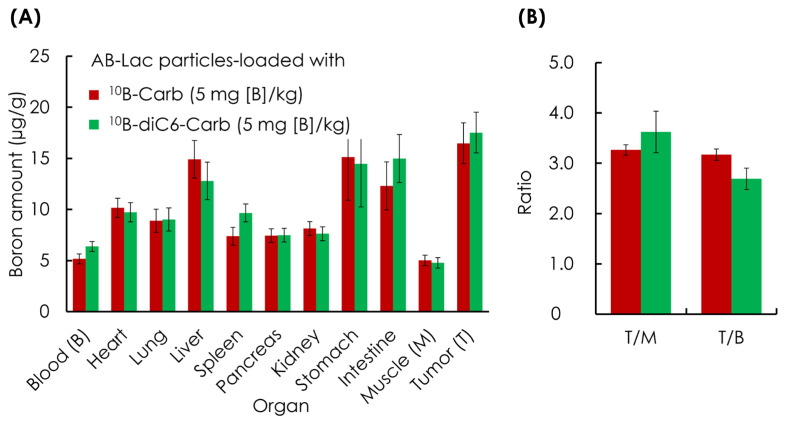
Ex vivo B biodistribution of AB-Lac particles-loaded with a ^10^B compound in the xenografts. Biodistribution of B amount in different organs (**A**) and T/M and T/B ratio at 24 h post-injection (**B**). Data are represented as mean ± S.E.M. (*n* = 4).

At 24 h post-injection of AB-Lac particles-loaded with ^10^B-diC6-Carb, 18 µg/g as well as those loaded with ^10^B-Carb, 16 µg/g, a high ^10^B-accumulation was reached in the tumors (Figure 3A). Furthermore, the results also showed a remarkably high B accumulation also in the liver, stomach, and intestines compared with the muscle tissue. We interpret this observation to mean that AB-Lac particles-loaded with any ^10^B compound are metabolized by the liver and excreted through the intestines.

The tumor-to-muscle (T/M) ratio was approximately 3.0:1 for all treated xenografts. In detail, the measured T/M ratio in the group loaded with ^10^B-diC6-Carb was 3.6:1, and it was 3.3:1 in the group loaded with ^10^B-Carb. The tumor/blood (T/B) ratio in both B compound loaded groups was similar, around 3:1 (Figure 3B).

The results were quite similar to those previously reported using other xenografts inoculated with murine breast cancer cells (e.g., 4T1 cells) [17]. The ^10^B biodistribution and the T/M and T/B ratios obtained from both experiments showed good conditions for performing BNCT effectively on different types of cancer cells.

### 3.3. In Vivo and Ex Vivo NIRF Imaging: Biodistribution of AB-Lac Particles in AsPC-1 Cells-Xenografts

**Figure 4:**
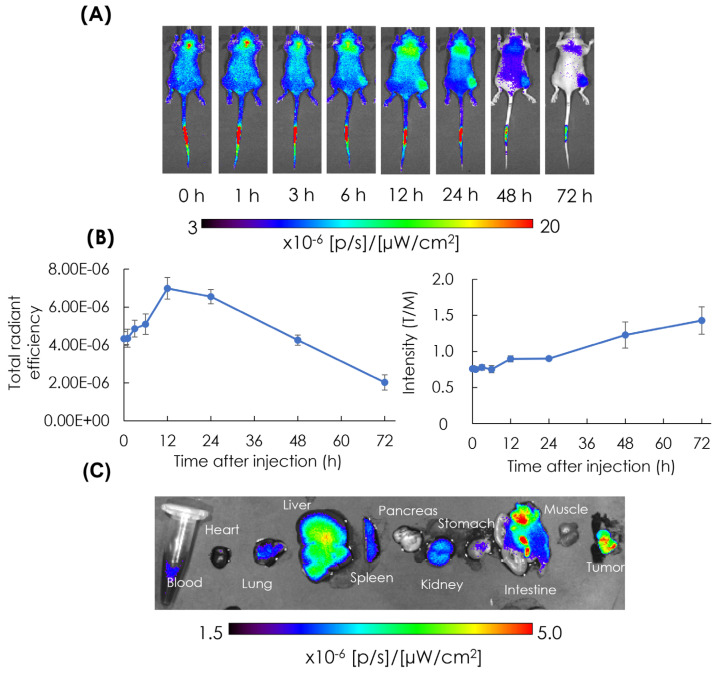
In vivo NIRF imaging in the AsPC-1 cells-xenografts. The representative NIRF imaging at each time point after the i.v. injection with ICG-labeled AB-Lac particles (**A**); the graph of fluorescence intensity in tumor lesions ((**B**); in the left panel) and the ratio of tumor intensity in muscle tissues (T/M ratio) in the xenografts injected with ICG-labeled AB-Lac particles ((**B**); in the right panel); ex vivo NIRF imaging of excised organs at 72 h post-injection (**C**). Data are represented as mean ± S.E.M. (*n* = 4).

The biodistribution of the particles in tumor lesions was observed in AsPC-1 cells-xenografts with the ICG-labeled AB-Lac particles injected i.v. via the tail vein. After that, in vivo NIRF imaging was performed at different time points from 1 to 72 h post-injection (Figure 4A).

ICG-labeled AB-Lac particles gradually accumulated in the tumor lesions and peaked at 12 h post-injection, as indicated by the intensity of their NIRF (Figure 4B, in the left panel). Furthermore, the T/M ratio gradually increased with the elapsed time, reaching the highest point 72 h after injection. (Figure 4B, in the right panel). These data show that ICG-labeled AB-Lac particles are distributed throughout the body approximately 1–3 h after injection.

After 72 h, the mice were euthanized and sacrificed. The organs, blood, heart, lung, liver, spleen, pancreas, kidney, stomach, intestine, muscle, and tumor were excised, and fluorescent intensity was quantified by ex vivo NIRF imaging (Figure 4C). The imaging showed that the liver had the highest intensity compared with muscle tissue, followed by the intestine and tumor. These results were previously reported using 4T1 tumor-bearing mice [17]. Another series of studies was conducted prior to a series of neutron irradiation studies with murine breast cancer cells (4T1 cells)-xenografts (Figure S1). The results showed that AB-Lac particles-loaded with ^10^B-Carb were found in the tumor lesions as was ^10^B-BPA.

### 3.4. In Vitro Cytotoxicity (Without Neutron Irradiation)

**Figure 5:**
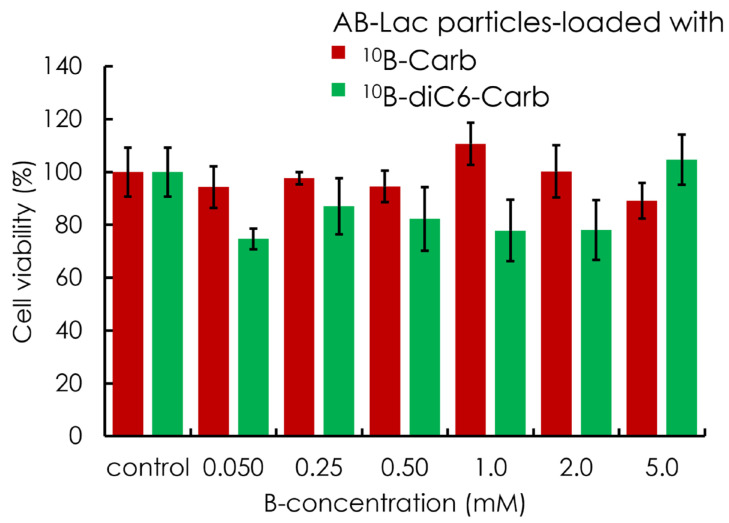
The in vitro cell viability with AB-Lac particles-loaded with ^10^B-Carb or ^10^B-diC6-Carb to AsPC-1 cells after 24 h incubation. The viability was observed by the CCK-8 kit. Data are represented as mean ± S.E.M. (*n* = 4).

The AsPC-1 cytotoxicity caused by AB-Lac particles-loaded with ^10^B-Carb or ^10^B-diC6-Carb was assessed. The cells were incubated with nanoparticles at 0.050, 0.25, 0.50, 1.0, 2.0, and 5.0 mM as the B-amount equivalent for 24 h. The cell viability was determined by the CCK-8 kit. The results indicated no significant cytotoxic effects in all groups, up to 5.0 mM of B concentration equivalent (Figure 5).

### 3.5. In Vitro Irradiation for BNCT

**Figure 6:**
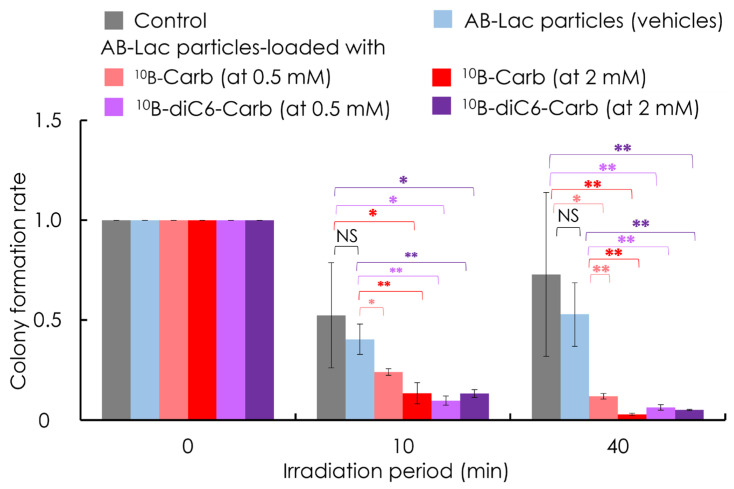
In vitro BNCT effect on AsPC-1 cell viability. Cells were treated with AB-Lac particles-loaded with ^10^B-Carb or loaded with ^10^B-diC6-Carb (at 0.5 mM or 2 mM of B-equivalent) for 2 h incubation, followed by irradiation at the KUR for 0, 10, and 40 min. The control group was treated with only medium and DPBS. AB-Lac particles (vehicles) included the same amount of AB-Lac polymer as AB-Lac particles with ^10^B-Carb (2 mM). After the irradiation, the treated cells were cultured for 14 days and then stained with 0.5% CV in 20% methanol. The graph shows the colony formation rate, and data are indicated as mean ± S.E.M. (*n* = 4). Significant differences are represented by ** *p* < 0.01 and * *p* < 0.05.

After 2 h incubation with AB-Lac particles-loaded with ^10^B-Carb or with ^10^B-diC6-Carb (at 0.5 mM and 2 mM of B-equivalent), the in vitro irradiation was performed. No significant effects were observed between the groups of “control” and “AB-Lac particles (vehicles)” with either 10 or 40 min irradiation (Figure 6). In contrast, it was obvious that at each time period of irradiation (for 10 and 40 min), treatments of AB-Lac particles-loaded with ^10^B-Carb or ^10^B-diC6-Carb significantly inhibited tumor growth. The inhibitory effect appeared to be irradiation time-dependent. Such effect was confirmed in similar experiments by comparing in vitro effects of ^10^B-BPA and ^10^B-Carb at 2 mM of B-equivalent in AsPC-1 cells (Figure S2).

### 3.6. In Vivo Irradiation for BNCT

**Figure 7:**
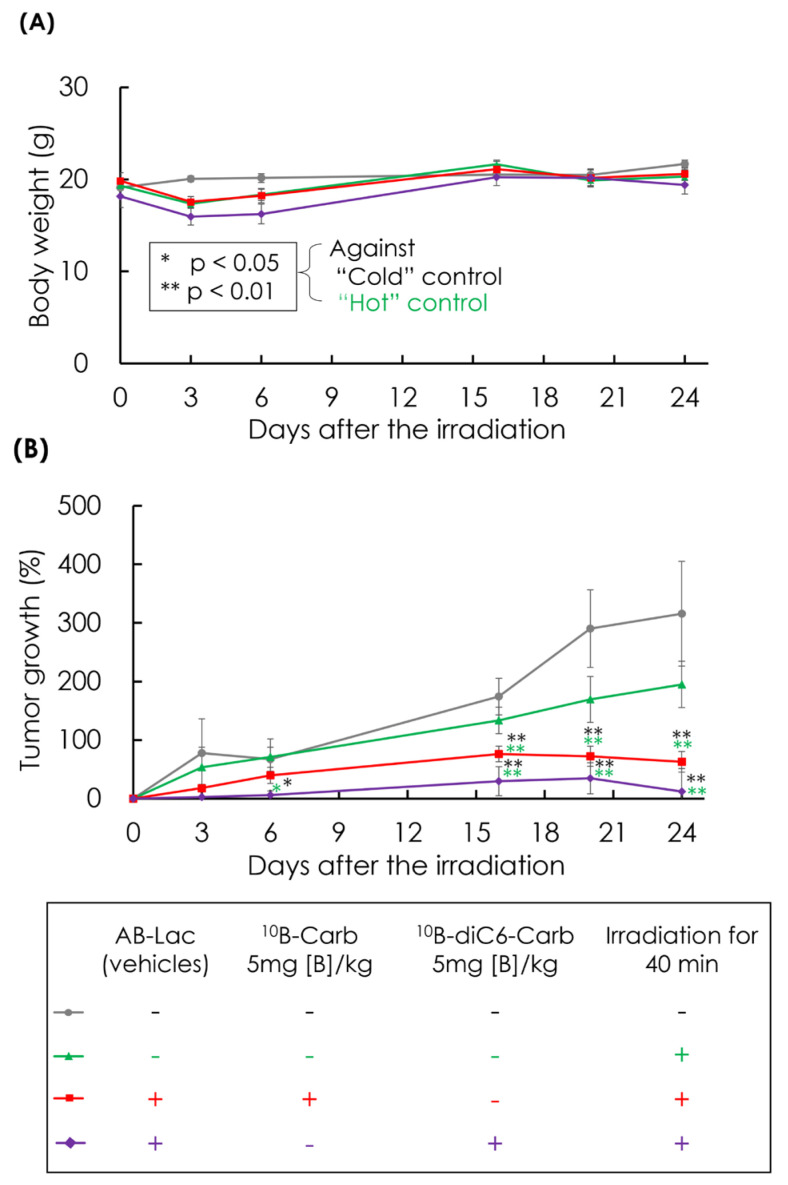
In vivo BNCT effect on tumor growth in the AsPC-1 cells-xenografts i.v. injected with AB-Lac particles-loaded with ^10^B-Carb or ^10^B-diC6-Carb (5 mg of B-equivalent/kg) at 24 h prior to the irradiation. The mice were irradiated by a KUR reactor at 5 MW for 40 min. Mice body weights (**A**) and the percentage of tumor growth (**B**) were evaluated until 24 days after the irradiation. Data are indicated as mean ± SEM (*n* = 4). Significant differences are represented by ** *p* < 0.01 and * *p* < 0.05.

The therapeutic effect (inhibitory effects on cancer growth) of the i.v. injection of AB-Lac particles-loaded with ^10^B-Carb or ^10^B-diC6-Carb (5 mg of B-equivalent/kg) was investigated. The in vivo irradiation did not alter the body weight of AsPC-1 cells-xenografts, and this means the irradiation by itself may not be physiologically toxic (Figure 7A). In both groups treated with AB-Lac particles-loaded with ^10^B-Carb and ^10^B-diC6-Carb, the tumor growth was significantly inhibited compared with the “cold” control and “hot” control (Figure 7B). It was shown that the BNCT effect was irradiation dose-dependent (Figure S3). Interestingly, the irradiation under the treatment of AB-Lac particles-loaded with ^10^B-diC6-Carb remarkably inhibited tumor growth in the xenografts compared with ^10^B-Carb.

### 3.7. In Vitro ICG-Dependent Inhibition of Cancer Growth

**Figure 8:**
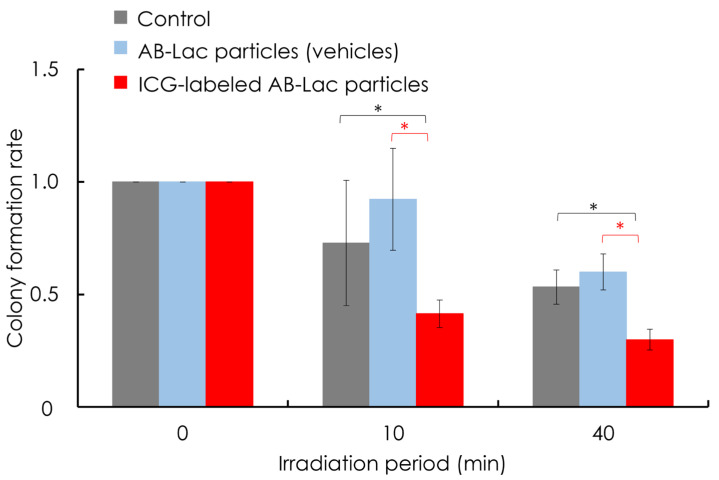
In vitro ICG-dependent irradiation effect on the AsPC-1 cell viability. The cultured AsPC-1 cells were treated with ICG-labeled AB-Lac particles for 2 h incubation, followed by irradiating by a KUR reactor at 1MW for 0, 10, or 40 min. After the irradiation, the cells were then incubated for 14 days and stained with 0.5% CV in 20% methanol. The graph shows the colony formation rate, and data are indicated as mean ± S.E.M. (*n* = 4). Significant differences are represented by * *p* < 0.05.

After the 2 h incubation with AB-Lac particles or ICG-labeled AB-Lac particles (without any ^10^B compounds), AsPC-1 cells were irradiated at the KUR reactor with 1 MW for 10 or 40 min. The cells were then cultured for 14 days and stained with CV. The results showed that ICG-labeled AB-Lac particles inhibited cancer cell proliferation compared with the control or AB-Lac particles, and the survival rate depended on the radiation dose. Cell proliferation was significantly inhibited by the irradiation. No significant effects were observed between the control (no particles) and AB-Lac particles (vehicles without boron-10) (Figure 8).

### 3.8. In Vivo ICG-Dependent Inhibition of Cancer Growth

**Figure 9:**
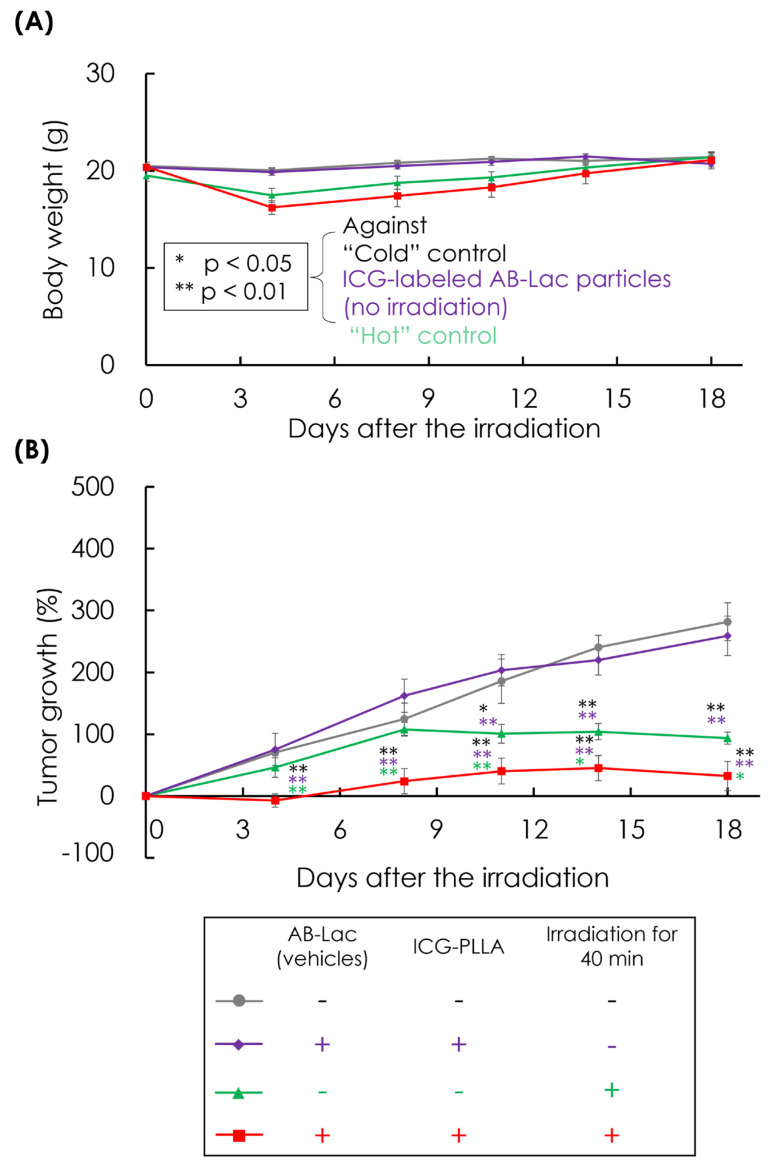
In vivo ICG-dependent irradiation effect on AsPC-1 cells-xenografts i.v. injected with ICG-labeled AB-Lac particles. The mice were irradiated by a KUR reactor at 5 MW for 40 min. Body weight (**A**) and percentage of tumor growth (**B**) were evaluated until 18 days after the irradiation. Data are indicated as mean ± S.E.M. (*n* = 4). Significant differences are represented by ** *p* < 0.01 and * *p* < 0.05.

We unexpectedly observed a cytotoxic effect similar to that of AB-Lac particles in AsPC-1 cells-xenografts that were infused with ICG-labeled AB-Lac particles (without ^10^B) under the same irradiation conditions. The body weight of the treated xenografts in all groups had not been changed by the irradiation (Figure 9A). Interestingly, the ICG-labeled AB-Lac particles group showed a significant inhibitory effect on tumor growth by the irradiation compared with the “cold” control, “hot” control, or “no irradiation” group until day 18 after the irradiation (Figure 9B). The significant therapeutic effect of irradiation under the treatment with ICG-labeled AB-Lac particles appeared from day 4.

## 4. Discussion

We have previously developed AB-Lac particles highly loaded with the hydrophobic ^10^B compounds ^10^B-Carb and ^10^B-diC6-Carb. The latter has a significant “molecular glue” effect [17]. In the present study, we performed neutron-irradiation studies with the nuclear reactor at the KUR.

The ICG labeling of AB-Lac particles was used as a fluorescence agent to observe the biodistribution of the particles [21]. Based on the results of this study, we have incidentally discovered two different cell death effects in cancer cells with our newly developed DDS nanoparticles (one through BNCT and one through ICG-dependent (but not BNCT) effects).

In the previous study without irradiation, we examined the ex vivo B biodistribution of AB-Lac particles-loaded with ^10^B-Carb or ^10^B-diC6-Carb in the xenografts 24 h after injection. The results showed that the B amount reached about 15–20 µg/g body weight, and the T/M and T/B ratios in the tumor were about 3:1, which is the minimum requirement for therapeutic effect in BNCT [22,23]. Furthermore, the biodistribution of the particles in AsPC-1 cells-xenografts and their accumulation in tumor lesions due to the “enhanced permeability and retention” effect (EPR effect) was observed by NIRF imaging [24].

The B accumulation in tumors peaked at 12 h post-injection. Regarding the metabolism of AB-Lac particles, the ex vivo NIRF imaging showed the highest intensity in the liver, followed by the intestines and the tumor lesions, which indicates a high level of excretion via the enterohepatic pathway [25]. The biodistribution pattern was quite similar (into liver and spleen) to most lipid-type nanoparticles. In contrast, the biodistribution of ^10^B compounds favored accumulation in the intestine as well as the liver (Figure 3). The explanation may be that hydrophobic B compound(s) alone, after dissociating from AB-Lac particles (due to the hydrolysis of the AB-Lac polymer in the liver), may be emulsified with bile acid and excreted into the intestine.

The ^10^B biodistribution in tumor lesions of 4T1 cells-xenografts was imaged ex vivo by LA-ICP-MS microscopy (Figure S1). This advanced technique can be used to observe specific sites where exogenous ^10^B, endogenous ^11^B, and ^31^P isotopes are concentrated, as previously reported by Reifschneider, et al. [26]. Our study showed that AB-Lac particles-loaded with ^10^B-Carb were accumulated in the tumor as was ^10^B-BPA, as previously reported [27]. Such preclinical screening is pivotal for ensuring the success of BNCT treatment, as enriched accumulation of ^10^B-isotopes, specifically in malignant lesions, is required.

In vitro and in vivo irradiation with neutrons for BNCT showed that AB-Lac particles-loaded with ^10^B-Carb and ^10^B-diC6-Carb achieved significant therapeutic (in vivo) effects. The inhibition of cancer cell growth was significant and took place in an irradiation time-dependent manner. Interestingly, the ^10^B compounds we developed, AB-Lac particles-loaded with ^10^B-Carb, showed an effective inhibitory effect (in vitro) at a short period (5 min) compared with the clinically used ^10^B compound, ^10^B-BPA (Figure S2). The results could also indicate that AB-Lac particles-loaded with ^10^B-diC6-Carb have great potential as a therapeutic tool for BNCT. These results support the “molecular glue” effect of ^10^B-diC6-Carb, as shown in our previous study [17].

We were able to achieve ^10^B-independent inhibitory effects on cancer cell viability in vitro and in vivo by treating with ICG-labeled AB-Lac particles compared with “cold” control groups, “hot” control groups, and non-irradiated groups. This effect appears to be caused directly by ICG alone. ICG is well known as a “theranostics” agent and is widely used for fluorescence, photothermal, and photosensitizing applications for minimally invasive cancer therapy such as photothermal therapy (PTT) [28], photodynamic therapy (PDT) [29,30], photoacoustic therapy [31], and ionizing radiation [32]. Although ICG shows great potential for treating hepatocellular carcinoma, gallbladder cancer, and gastric cancer in xenografts [29,30,33], there are still some challenges, such as its high susceptibility to degradation by laser irradiation, a complicated synthesis process, a short plasma half-life (about 3–4 min), and rapid excretion via enterohepatic pathways [34,35]. ICG labeled to nanoparticles and polymeric micelles, including Lactosome^®^, could solve these problems and improve its efficacy as a therapeutic and diagnostic modality [36,37,38].

**Figure 10:**
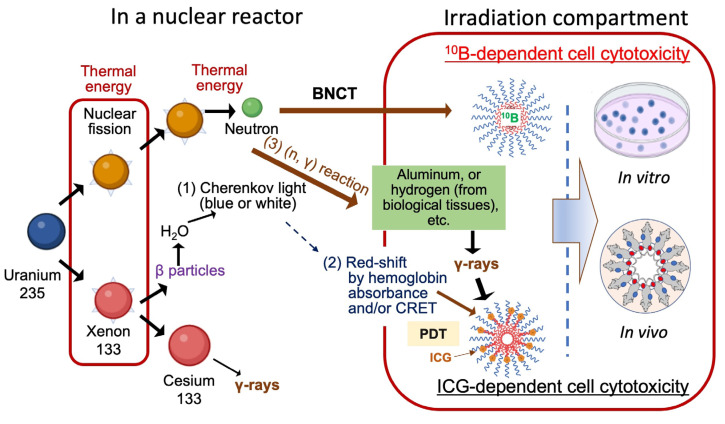
Schematic illustration of irradiation of B compound-loaded AB-Lac particles for BNCT effect and ICG-labeled AB-Lac particles for non-BNCT effect. This schematic illustration concept was adapted from the Ministry of the Environment, Government of Japan and the National Institutes for Quantum Science and Technology, etc. [39,41,44,45,47,51,53].

The dual effects observed in our irradiation study can be explained by the generation of nuclear fission products in a reactor facility. First, in the nuclear reactor, bombarding enriched uranium fuel (uranium-235) with neutrons results in nuclear fission reaction products, including neutrons that enable BNCT and other radiation qualities, such as α-particles, β-particles, X-rays, and γ-rays (Figure 10) [39]. This complex radiation field interacts with materials, creating ionization and nuclear reactions that in turn generate other particles and electromagnetic waves [40,41].

For the ^10^B-independent effect, one possibility could be a PDT effect that might occur due to Cherenkov radiation (CR)-induced PDT (CR-PDT), where therapeutic effect is strongly influenced by β-particles and photosensitizers such as ICG [42,43] (Figure 10). However, since Cherenkov light represents less than 0.006% of the total energy released during radioactive decay in a nuclear reactor [44,45], significant therapeutic effects from Cherenkov light may be achievable with the presence of 6800 OH radicals, which play a key role in mediating the biological action of ionizing radiation on DNA [45]. Therefore, there is little possibility that this is the cause of the observed therapeutic effects.

The second possibility for the ^10^B-independent effects is the activation of ICG by a wavelength shift of CR in the biological tissue. CR is located at visible light at 300–600 nm, while the Cherenkov emission spectrum in mammalian tissues would be dominant at the wavelength 600–900 nm due to the high absorption of short wavelengths by the blood [46,47,48,49]. In these steps, CR-PDT might occur with the presence of fluorophores such as ICG (excitation at 750 nm), which could be activated through a small portion of CR toward the near-infrared window by Cherenkov radiation energy transfer (CRET) and photogenerated reactive oxygen species (ROS) resulting in therapeutic effects [37,46,50,51]. In our study, ICG could act as a photosensitizer and trigger photodynamic and/or photothermal effects [35,51].

The third possibility is the direct activation of ICG by γ-rays, which may affect cancer cell viability and are generated by (n, γ) reaction by interaction with infrastructural metals like the aluminum of the irradiation compartment or hydrogens in biological tissues, etc. In this case, ICG might act as a sensitizer to γ-rays, inducing cell death by photoactivation. Cytotoxic damage could be due to the fact that ionizing radiation of the sensitizer induces the formation of ROS, which in turn cause mitophagy and DNA damage [52,53]. The combination of a photosensitizing agent and ionizing radiation has been previously reported by several groups. Ben-Hur, et al. reported the combination of chloroaluminum phthalocyanine tetrasulfonate and γ-rays in Chinese hamster cells and human lymphocytes [54]. Tsukanishi, et al. revealed the effective antitumor effects of ICG-lactosomes when combined with laser irradiation on a human breast cancer cell line [55]. Montazerabadi, et al. demonstrated the inhibition effects of ICG on a human breast cancer cell line with X-rays as ionizing radiation [52]. Clement, et al., demonstrated X-rays induced singlet oxygen generation by nanoparticle–photosensitizer conjugates [56]. The influence of ionizing radiation, whether γ-rays or Χ-rays, may affect therapeutic outcomes when combined with photosensitizing agents, and Cherenkov light may not trigger the activation of photosensitizing agents [57]. In any case, the effect of the specific type of ionizing radiation used in our study with ICG-attached AB-Lac particles merits further study.

Previous observations of dual effects in BNCT have been reported by Friso, et al. on the combination of ^10^B-enriched Carb containing phthalocyanine and by Hiramatsu, et al. on the dual BNCT and PDT agent Tetrakis (*p*-carboranylthiotetrafluorophenyl) chlorin (TPFC) for BNCT and PDT applications [58,59]. Therefore, we expect that our ICG-attached AB-Lac particles and AB-Lac particles-loaded with ^10^B compound(s) may be a novel and promising candidate, providing not only NIRF imaging for practical diagnosis but also therapeutic effects on the tumor tissues.

Despite the promising results from the current irradiation study, we are planning to develop other formulations to obtain a higher boron concentration by adjusting the proportion of AB-Lac particles and ^10^B compounds carrying ICG. We have also observed other types of amphipathic polymer DDSs, that is, A_3_B-Lac particles composed of three hydrophilic PSars and a hydrophobic PLLA with 22 nm size. Lim, et al. reported that the A_3_B-Lac conjugated with small interfering RNA (siRNA) exhibited a relatively lower EPR effect on tumor lesions [60,61]. Moreover, a 27 kDa human single chain variable fragment (scFv) of IgG against the mesothelin (MSLN) and cell-penetrating peptides conjugated with AB or A_3_B-Lac particles may be effective to enhance selectivity and penetration in tumors, [10,62]. We expect that our future work could enhance the therapeutic effect of both AB- and A_3_B-Lac particles loaded with ^10^B-Carb or with ^10^B-diC6-Carb for dual cancer therapy.

## 5. Conclusions

We demonstrated the inhibitory effects of AB-Lac particles-loaded with hydrophobic ^10^B compounds and ICG-labeled AB-Lac particles on cancer cell growth. ^10^B-biodistribution showed, ex vivo, a sufficient amount of boron and good T/M and T/B ratios for a successful BNCT. In vivo and ex vivo NIRF imaging confirmed the EPR effect occurred in ICG-labeled AB-Lac particles, reaching the peak intensity at 12 h post-injection. The imaging of excised tumors and organs showed that the liver had the highest intensity. Irradiation studies conducted both in vitro and in vivo showed that AB-Lac particles-loaded with the ^10^B compounds were effective in inhibiting the growth of AsPC-1 cancer cells. Interestingly, a non-BNCT effect was achieved in the group treated with ICG-labeled AB-Lac particles in vitro and in vivo. The data obtained indicate ways to further develop ICG-labeled AB-Lac particles-loaded with a hydrophobic ^10^B compound as novel particles for cancer theranostics.

## Data Availability

All data that were generated for this study are available upon reasonable request.

## Supplementary Materials

The following supporting information can be downloaded at: www.mdpi.com/article/10.3390/cells14010060/s1, Figure S1. Ex vivo LA-ICP-MS imaging of ^10^B-biodistribution in tumor lesions of the 4T1 cells-xenografts. Figure S2. In vitro irradiation of AsPC-1 cells for BNCT [63]. Figure S3. In vivo irradiation of tumor lesions in the AsPC-1 cells-xenografts for BNCT.
