# Supplementary material for: Novel Drug Delivery Particles Can Provide Dual Effects on Cancer “Theranostics” in Boron Neutron Capture Therapy
Source: Cells. 2025 Jan 6;14(1):60. doi: 10.3390/cells14010060 (PMC11719788; doi:10.3390/cells14010060)
Supplement: Supplementary file 1 [file cells-14-00060-s001.zip › cells-3360700-supplementary.pdf]

Supporting information

Novel Drug Delivery Particles Can Provide Dual Effects on Cancer "Theranostics" in Boron Neutron Capture Therapy

Abdul Basith Fithroni[1], Haruki Inoue[1], Shengli Zhou[1], Taufik Fatwa Nur Hakim[1], Takashi Tada[1], Minoru Suzuki[2], Yoshinori Sakurai[2], Manabu Ishimoto[3], Naoyuki Yamada[4], Rani Sauriasari[5], Wolfgang A.G. Sauerwein[6], Kazunori Watanabe[1], Takashi Ohtsuki[1], and Eiji Matsuura[1,5,7,8*]

[1] Graduate School of Interdisciplinary Science and Engineering in Health Systems, Okayama University, 700-8530, Okayama, Japan; abdul-bf@s.okayama-u.ac.jp (A.B.F.); kako122232@s.okayama-u.ac.jp (H.I.); p0ux6145@s.okayama-u.ac.jp (S.Z.); pmcx89by@s.okayama-u.ac.jp (T.F.N.H.); pv6s2ev1@s.okayama-u.ac.jp (T.T); ohtsuk@okayama-u.ac.jp (T.O.); eijimatu@md.okayama-u.ac.jp (E.M.)

[2] Institute for Integrated Radiation and Nuclear Science, Kyoto University, 590-0494, Osaka, Japan; suzuki.minoru.3x@kyoto-u.ac.jp (M.S.); sakurai.yoshinori.8n@kyoto-u.ac.jp (Y.S.)

[3.] J-BEAM, Inc., 979-0513, Fukushima, Japan; manabu.ishimoto@gmail.com (M.I.)

[4.] Nihon Fukushi Fuiin Holding, Co., Ltd., 979-0513, Fukushima, Japan; naoyuki.yamada@fukushi-chuho.com (N.Y.)

[5.] Faculty of Pharmacy, Universitas Indonesia, 16424, Depok, Indonesia; rani@farmasi.ui.ac.id (R.S.); (E.M.)

[6.] Deutsche Gesellschaft für Bor-Neutroneneinfangtherapie DGBNCT e.V., postal address: University Hospital Essen, Klinik für Strahlentherapie, 45122, Essen, Germany; wolfgang.sauerwein@dgbnct.de (W.A.G.S.)

[7] Collaborative Research Center for OMIC, Graduate School of Medicine, Dentistry, and Pharmaceutical Sciences, Okayama University, 700-8558, Okayama, Japan (E.M.)

[8] Neutron Therapy Research Center (NTRC), Okayama University, 700-8558, Okayama, Japan (E.M.)

* Correspondence: Eiji Matsuura, Ph.D. (E.M.); eijimatu.01@gmail.com and eijimatu@md.okayama-u.ac.jp; Tel.: +81-86-251-8088. Mailing address: Graduate School of Interdisciplinary Science and Engineering in Health Systems, Okayama University, 700-8530, Okayama, Japan

List of contents

Figure S1. ***Ex vivo*** **LA-ICP-MS imaging of ^{10}B-biodistribution in tumor lesions of 4T1 cells-xenografts.**
Figure S2. ***In vitro*** **irradiation of AsPC-1 cells for BNCT.**
Figure S3. ***In vivo*** **irradiation of tumor lesions in AsPC-1 cells-xenografts for BNCT.**

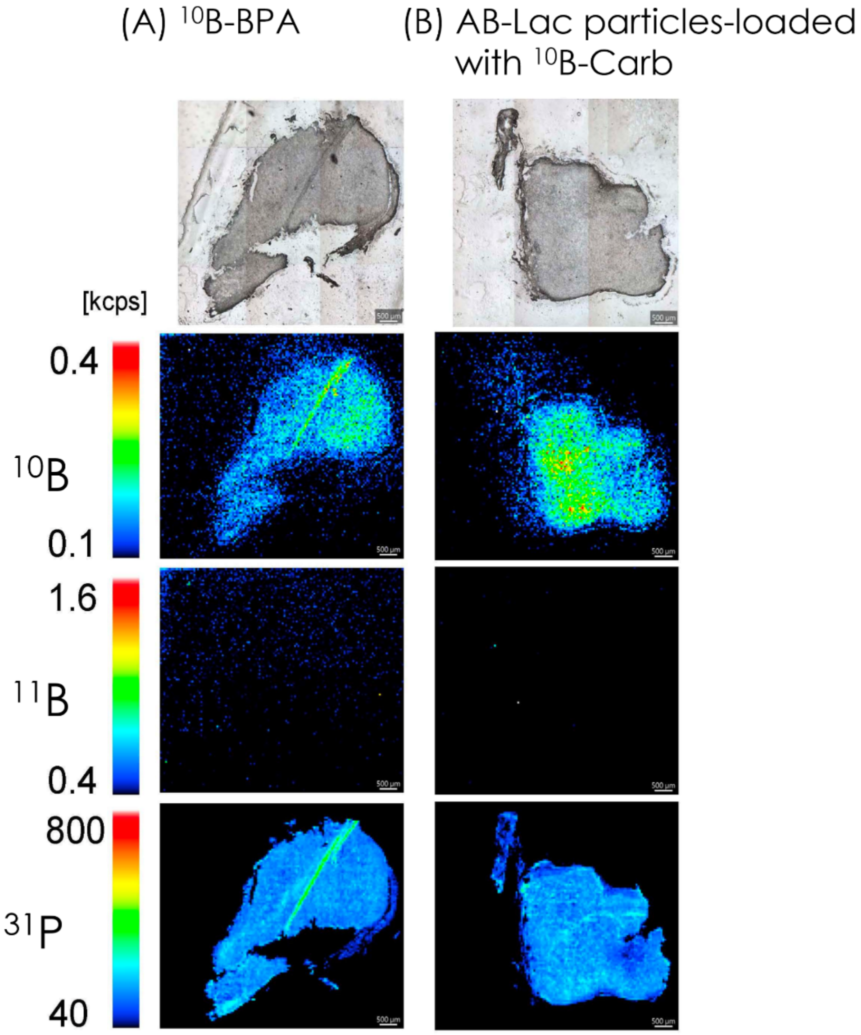

Figure S1. ***Ex vivo*** **LA-ICP-MS imaging of ^{10}B-biodistribution in tumor lesions of 4T1 cells-xenografts.** Murine breast cancer cells (4T1 cells; 1×10^{6} cells) were subcutaneously (s.c.) inoculated in the right thigh of the mice two weeks before the i.v. injection of ^{10}B-BPA **(A)**; and AB-Lac particles-loaded with ^{10}B-Carb **(B)** at respective B-amount of 5 mg /kg. At 6 h post-injection, the xenografts were euthanized and the tumor legions were excised. The image of lesional tumor sections were embedded in Tissue-Tek® O.C.T. compound (Sakura Finetek Japan, Co., Ltd., Tokyo, Japan) , frozen at -80 °C, and sliced to 50 µm thickness. The imaging views were obtained by laser ablation-inductively coupled plasma-mass spectrometry (LA-ICP-MS; LSX-213 G2^{+} and ICPMS-2030, Shimadzu).

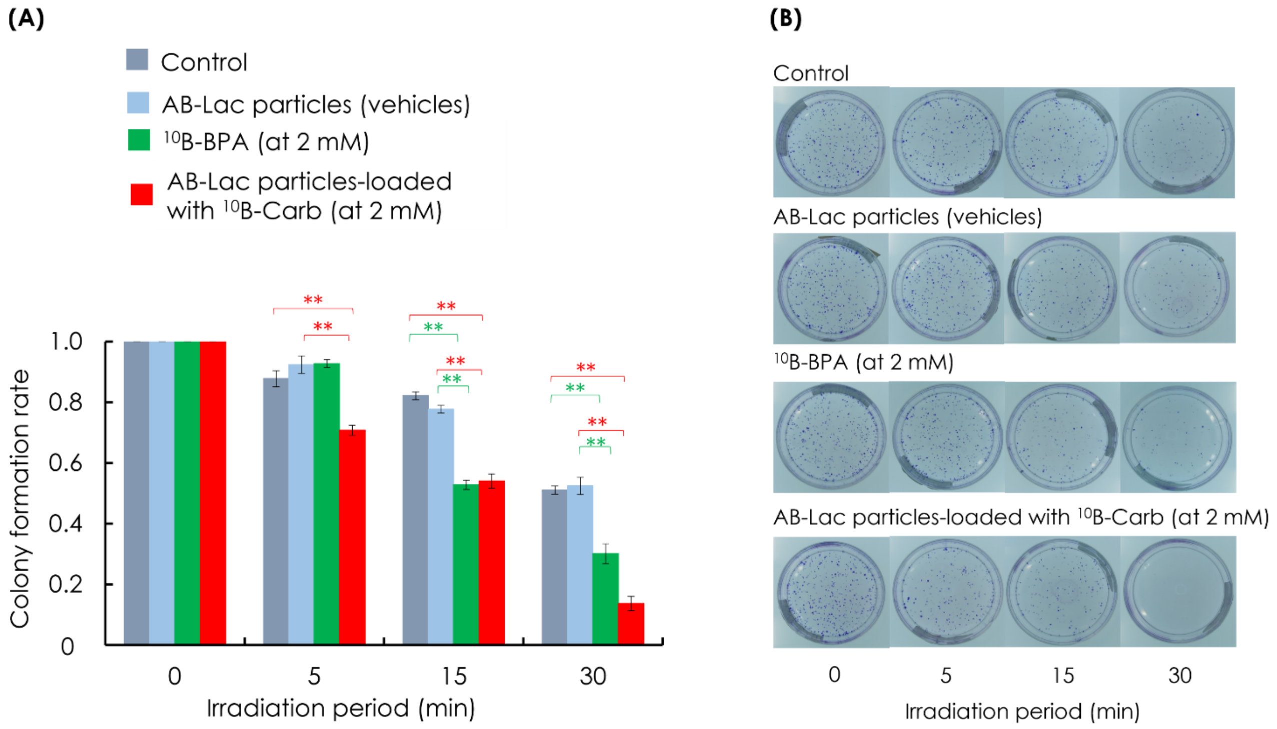

Figure S2. *In vitro* irradiation of AsPC-1 cells for BNCT. For assessing the BNCT effect by *in vitro* irradiation, the cultured AsPC-1 cells were treated with ^{10}B-BPA and AB-Lac particles-loaded with ^{10}B-Carb (at 2 mM of B-equivalent) for 2 h, followed by irradiation for 0, 5, 15, and 30 min **(A)**. The cells were then irradiated by a KUR reactor at 1 MW for 5 min (thermal neutron fluence: 6.2×10^{11} neutrons/cm^2, γ-ray dose: 0.076 Gy), for 15 min (thermal neutron fluence: 8.9×10^{11} neutrons/cm^2, γ-ray dose: 0.14 Gy), and for 30 min (thermal neutron fluence: 3.5×10^{12} neutrons/cm^2, γ-ray dose 0.58 Gy), respectively. After the irradiation, the cells were again cultured for 14 days, then stained using 0.5% CV in 20% methanol. ^{10}B-BPA solution was prepared as the methods reported by Coderre, *et al*. [1] In brief, ^{10}B-BPA (90 mg/mL) and fructose (75 mg/mL) was dissolved in water to obtain B^{10}-BPA-fructose solution (at a concentration of 0.42 M of ^{10}B-BPA). The solution was then stirred gently to adjust pH 9.5-10. After starring for a couple of minutes, pH was adjusted to 7.4 with HCl and the solution was sterilized by a 0.22 μm-filter. The graphs show the colony formation rate **(A)** and the representative image **(B)**. Data are indicated as mean ± S.E.M. (n = 4). Significant differences are represented by $^{**}p < 0.01$ and $^{*}p < 0.05$.

References

1. Coderre, J.A.; Button, T.M.; Micca, P.L.; Fisher, C.D.; Nawrocky, M.M.; Liu, H.B. Neutron capture therapy of the 9l rat gliosarcoma using the p-boronophenylalanine-fructose complex; 1994; Int J Radiat Oncol Biol Phys 1994 Oct 15;30(3):643-52. doi: 10.1016/0360-3016(92)90951-d

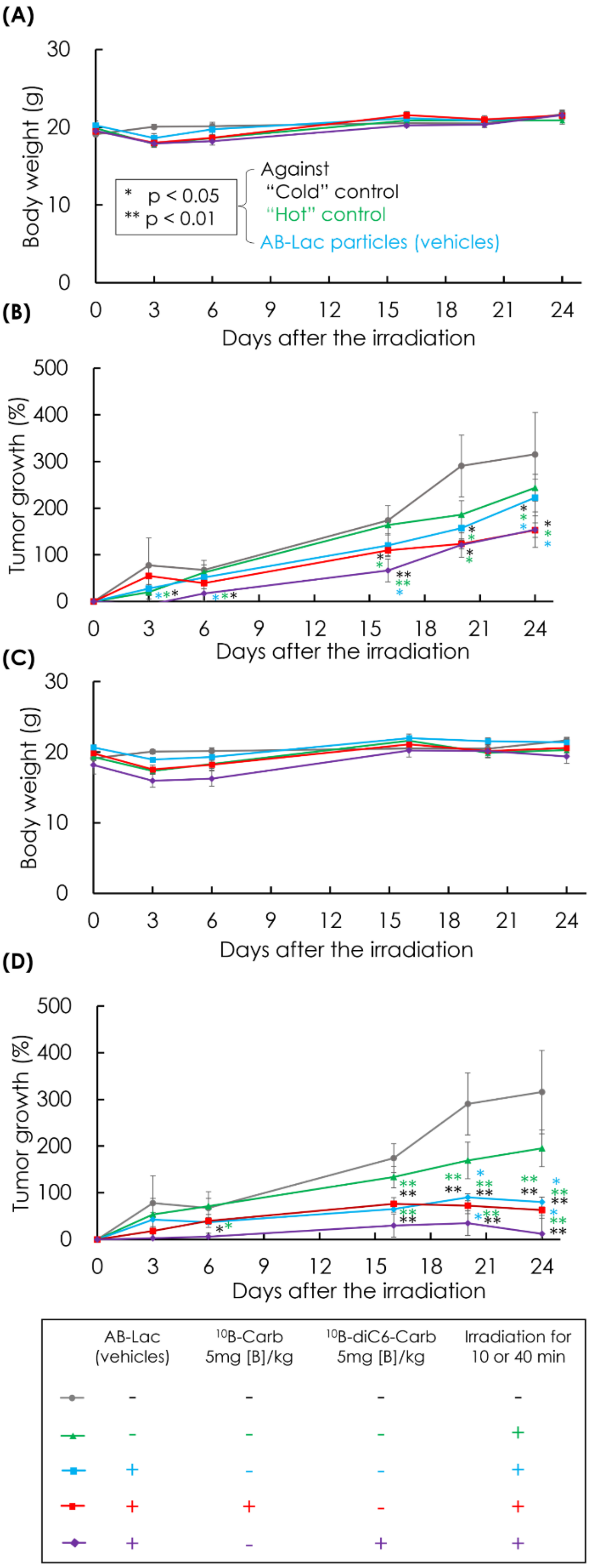

Figure S3. ***In vivo*** **irradiation of tumor lesions in As-PC-1 cells-xenografts for BNCT.** For assessment of *in vivo* irradiation, the AsPC-1 xenografts were i.v. injected with AB-Lac particles (vehicles), and those loaded with ^{10}B-Carb or with ^{10}B diC6-Carb (5 mg of ^{10}B-equivalent/kg), respectively. The irradiation was performed by a KUR rector at 5 MW for 10 min (thermal neutron fluence: $2.5{\times}10^{12}$ neutrons/cm^2, γ-ray dose: 0.33 Gy) or for 40 min (thermal neutron fluence: $9.4{\times}10^{12}$ neutrons/cm$^-$, γ-ray dose: 1.2 Gy), respectively. Body weight and tumor growth were monitored on day 24th after the irradiation. Body weight **(A)** at 10min and **(C)** at 40 min and the percentage of tumor growth **(B)** at 10 min and **(D)** at 40 min. Data are indicated as mean ± SEM (n=4). Significant differences are represented by $^{**}p < 0.01$ and $^{*}p < 0.05$.
